# Estimation of the Health Impact and Cost-Effectiveness of Influenza Vaccination with Enhanced Effectiveness in Canada

**DOI:** 10.1371/journal.pone.0027420

**Published:** 2011-11-14

**Authors:** David N. Fisman, Ashleigh R. Tuite

**Affiliations:** Division of Epidemiology, Dalla Lana School of Public Health, University of Toronto, Toronto, Ontario, Canada; University of Hong Kong, Hong Kong

## Abstract

**Introduction:**

The propensity for influenza viruses to mutate and recombine makes them both a familiar threat and a prototype emerging infectious disease. Emerging evidence suggests that the use of MF59-adjuvanted vaccines in older adults and young children enhances protection against influenza infection and reduces adverse influenza-attributable outcomes compared to unadjuvanted vaccines. The health and economic impact of such vaccines in the Canadian population are uncertain.

**Methods:**

We constructed an age-structured compartmental model simulating the transmission of influenza in the Canadian population over a ten-year period. We compared projected health outcomes (quality-adjusted life years (QALY) lost), costs, and incremental cost-effectiveness ratios (ICERs) for three strategies: (i) current use of unadjuvanted trivalent influenza vaccine; (ii) use of MF59-adjuvanted influenza vaccine adults ≥65 in the Canadian population, and (iii) adjuvanted vaccine used in both older adults and children aged < 6.

**Results:**

In the base case analysis, use of adjuvanted vaccine in older adults was highly cost-effective (ICER = $2111/QALY gained), but such a program was “dominated” by a program that extended the use of adjuvanted vaccine to include young children (ICER = $1612/QALY). Results were similar whether or not a universal influenza immunization program was used in other age groups; projections were robust in the face of wide-ranging sensitivity analyses.

**Interpretation:**

Based on the best available data, it is projected that replacement of traditional trivalent influenza vaccines with MF59-adjuvanted vaccines would confer substantial benefits to vaccinated and unvaccinated individuals, and would be economically attractive relative to other widely-used preventive interventions.

## Introduction

Influenza is a contagious acute respiratory disease that is responsible for an estimated 4000 deaths annually in Canada, due both the influenza and its downstream complications [1], with deaths mainly occurring in adults aged 65 and older. Although most influenza infections are self-limiting, they result in increased demands on health care services and are costly in terms of morbidity and lost productivity [2], [3], [4], [5].

When the vaccine is well matched with circulating influenza strains, immunization is an effective preventive measure for reducing influenza-attributable morbidity and mortality. Unadjuvanted trivalent influenza vaccine (TIV), containing three specific subtypes of influenza expected to dominate during the upcoming influenza season (two influenza A strains and one influenza B strain), is currently used in Canada. The composition of the vaccine is updated annually to reflect changes in the dominant circulating subtypes, due to antigenic drift or antigenic shift.

Efficacy of unadjuvanted vaccine in older adults (≥65) is typically lower than that observed in healthy adults [6]; this reduced efficacy may be due to a lowered antibody response to the vaccine in the elderly [7]. To overcome this reduced efficacy, influenza vaccines containing an adjuvant to enhance immune response have been used in older adults in some European countries [8]. Additionally, during the recent pH1N1 pandemic, adjuvanted vaccine was adopted as an antigen-sparing measure by many countries, where its use was not restricted to older adults. In the elderly and young children, there is emerging evidence that adjuvanted trivalent influenza vaccines (ATIV) result in enhanced protection against influenza infection or adverse outcomes following infection [9], [10], [11]. It has also been proposed that these vaccines may provide protection against viral drift, thereby enhancing the duration of immunity against infection [12], [13], [14].

Given the evidence of both enhanced vaccine efficacy and enhanced duration of immunity associated with ATIV, we sought to evaluate the effect of using a seasonal adjuvanted vaccine in the Canadian population. We used an age-structured mathematical model to evaluate the impact of seasonal influenza vaccination on expected influenza transmission over a 10-year period. Model projections were used to perform an economic evaluation to estimate projected health outcomes and costs associated with the use of adjuvanted vaccine compared to the currently used unadjuvanted vaccine in the Canadian population.

## Methods

### Model Construction

**Figure 1:**
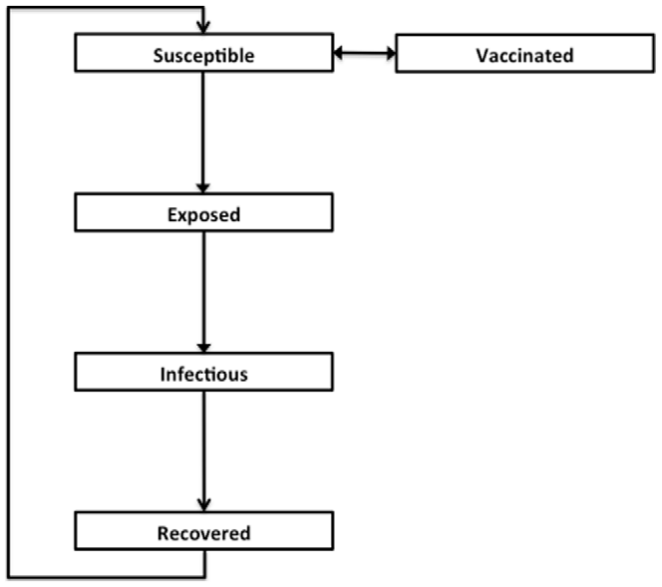
Outline of model structure, showing population flows between compartments. Each compartment is further stratified by age category.

We constructed an age-structured compartmental model that simulates the transmission of influenza in the Canadian population, as described in detail in [15], [16]; this model was modified to include births and non-influenza deaths, in order to examine multi-year influenza dynamics. The model structure is presented in **Figure 1** and additional model details are provided in **File S1**. Natural history assumptions (**Table 1**) were derived from epidemiologic studies and by model calibration. The population was divided into five compartments representing different disease states: susceptible (S), vaccinated (V), exposed (E; i.e., infected but not infectious), infectious (I), and recovered (R).

**Table 1:** Transmission model parameter values.

Variable	Age group	Value (range)	Source
Total population size	All	31,612,905	[44]
Life expectancy (years)	All	75.3	[45]
Latent period (days)	All	2.5	[46]
Duration of infectiousness (days)	All	3.5	[46]
Basic reproductive number	All	1.6 (1.4–1.9)	Model calibration
Duration of immunity (years)			Model calibration and assumption
Following infection	All	1.3 (1–2)	
Following vaccination	All	1 (1–2)	
Proportion vaccinated	<1	0.12	[17], [47]
	1–5	0.28	
	6–19	0.30	
	20–64	0.33	
	≥65	0.75	
Vaccine efficacy			[9], [10], [19], [20], [21] and model calibration
Trivalent influenza vaccine (TIV)	<6	0.5 (0–0.83)	
	6–64	0.9 (0.7–0.9)	
	≥65	0.2 (0–0.2)	
Adjuvanted influenza vaccine (ATIV)	<6	0.9 (0–0.9)	
	≥65	0.4 (0.2–0.4)	
			

Vaccination was modeled by removing individuals from the susceptible compartment during a three-month period each year, beginning approximately 4 months prior to peak influenza activity. The model was calibrated to reproduce average excess seasonal influenza-attributable mortality rates observed in the Canadian province of Ontario over seven influenza seasons (1997–2004) [17].

### Vaccine Uptake and Strategies

Ontario introduced a Universal Influenza Immunization Program (UIIP) in 2000, which theoretically removes barriers to vaccination in the population. As this program has been projected to be cost-effective in the Canadian context [18] our base-case analysis included immunization with TIV for individuals aged 6–64 at rates observed in the Ontario UIIP. We regarded rates of vaccine uptake observed in the UIIP as those expected with ATIV. Vaccine efficacy estimates were derived from trials and observational studies for ATIV, and from both published estimates and model calibration for TIV [9], [10], [11], [19], [20], [21]. Approaches to estimates of relative efficacy are presented in **File S1**.

We assumed the population was immunized at UIIP rates. Individuals aged 6–64 were immunized with TIV, with an efficacy of 0.9 in all scenarios. We evaluated three strategies: (i) immunization of children and older adults with TIV; (ii) immunization of children with TIV and older adults with ATIV; and (iii) immunization of children and older adults with ATIV. We repeated the same scenarios in the absence of vaccination in the 6–64 age group.

### Estimation of Burden of Disease and Costs

The age-specific impact of influenza on healthcare utilization and cost was estimated using the approach of Sander et al. [18], [22] and based on event probabilities as described by Kwong et al. [17]. Details are presented in **File S1**, and costs are presented in **Table 2** and **File S1**. A ten-year time horizon was used in the analysis and we did not include pandemic years in the analysis.

**Table 2:** Parameter values used in the economic evaluation.^a^

	Age group	Value (range)	Source
Total costs per infection ($)			[18]
	0–5	13.76 (3.56–86.17)	
	6–19	8.30 (2.33–33.21)	
	20–64	11.33 (2.59–63.50)	
	≥65	23.85 (4.37–165.57)	
Total cost per vaccine dose ($)			
Trivalent influenza vaccine	All	7.55	[18]
Adjuvanted	All	11.59 (8.59–18.59)	[22]
QALY lost per influenza infection			[18]
	0–5	0.015 (0.0065–0.022)	
	6–19	0.015 (0.0065–0.022)	
	20–64	0.017 (0.0097–0.025)	
	≥65	0.029 (0.023–0.035)	
QALY lost per death due to influenza (discounted at 5%)			[22]
	0–5	18.530	
	6–19	18.150	
	20–64	15.140	
	≥65	2.410	
Discount rate (%)	All	5.0	[39]

The range indicates the minimum and maximum values used in sensitivity analyses.

a Additional details provided in Table S1.

### Sensitivity Analyses

To determine the sensitivity of our base case findings to assumptions around the costs and consequences of influenza infection and vaccine costs, we conducted a one-way sensitivity analysis, with parameters varied one at a time across the range of plausible values outlined in **Table 2** and **File S1**. We also calculated ICERs for best and worst case sets of parameters (i.e., simultaneously setting all parameters to their extreme values).

Given the uncertainly surrounding vaccine efficacy (for both TIV and ATIV) and model assumptions, we conducted sensitivity analyses to determine the robustness of our base case findings. We estimated the vaccine efficacy values at which use of ATIV was no longer cost effective for different willingness-to-pay thresholds (ranging from $1000 to $50,000 per QALY).

In base case analyses, we assumed that the duration of immunity to influenza infection was 1.3 years following natural infection and 1 year following vaccination. It has been suggested that immunization with adjuvanted vaccine results in enhanced duration of immunity, due to the induction of a broader immune response than that observed with TIV [23]. We assessed the impact of enhanced durability of immunity following vaccination (up to 2 years) with ATIV.

## Results

### Model Calibration

Model projected estimates of average influenza-attributable mortality were comparable to those observed in Ontario, assuming reported UIIP vaccination rates (**Figure S1**). Because the model assumed constant influenza transmissibility and vaccine efficacy over time, it did not reproduce the observed year-to-year variability in influenza incidence and mortality.

### Enhanced Vaccine Efficacy with Adjuvanted Vaccine

**Figure 2:**
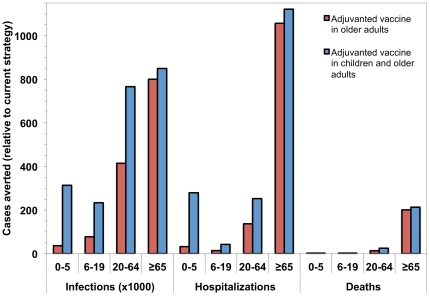
Projected health benefits of using adjuvanted influenza vaccine. Health benefits are estimated for a strategy in which adults >65 or adults ≥65 and children <6 years are vaccinated with adjuvanted influenza vaccine. Projected number of infections, hospitalizations, and deaths averted, by age, over a 10-year period were calculated relative to the use of unadjuvanted trivalent influenza vaccine in the entire population over this time period.

Use of ATIV in children under 6 and adults aged ≥65 in the Canadian population, with continued use of TIV in the population aged 6–64, was projected to provide substantial health benefits, including aversion of deaths and hospitalizations, relative to currently used TIV (**Figure 2**). In the base case analysis, use of ATIV in adults aged ≥65 was highly cost effective, with an incremental cost-effectiveness ratio (ICER) of $2111 per QALY gained, relative to use of TIV. While the cost of using ATIV was substantially higher than TIV ($837.0 versus $730.5 million over 10 years), part of this cost was offset by reducing the number of cases, and consequently, health care resource use due to influenza treatment, from $501.76 to $473.50 million. Expanding ATIV coverage to include young children weakly dominated the strategy that included vaccination of older adults only, with an ICER of $386 per QALY gained. As such, a program that covered both young children and older adults with ATIV would be preferred to one that covered only older adults, with an ICER of $1612 per QALY. Discounted costs and benefits, and incremental cost-effectiveness ratios for the alternate strategies are shown in **Table 3**.

**Table 3:** Incremental cost-effectiveness of influenza vaccination strategies targeting children and older adults implemented in the Canadian population: base case, with trivalent influenza vaccination in individuals aged 6–64.

Strategy	Vaccine efficacy	Cost ($ billion)^a^	QALY lost (million)^b^	Incremental cost per QALY gained ($)
Immunization with TIV	0.5 in children; 0.9 in persons 6–64; 0.2 in older adults	1.232	0.749	–
Immunization of children and persons aged 6–64 with TIV and older adults with ATIV	0.5 in children; 0.9 in persons 6–64; 0.4 in older adults	1.310	0.712	Weakly dominated^c^
Immunization of children and older adults with ATIV and persons 6–64 with TIV	0.875 in children; 0.9 in 6–64; 0.4 in older adults	1.316	0.697	1612

Abbreviations: TIV, trivalent inactivated vaccine; ATIV, adjuvanted trivalent inactivated vaccine.

a 2009 Canadian dollars, discounted at 5% annually over a 10-year time horizon.

b Quality-adjusted life years lost, discounted at 5% annual over a 10-year time horizon.

c Immunization of older adults only with ATIV was economically attractive at $2111 per QALY, but the incremental cost-effectiveness ratio of immunizing *both* older adults and young children with ATIV was <$500 per QALY, indicating “extended dominance”.

Qualitatively similar results were observed when the scenarios were repeated excluding immunization of the population aged 6–64, with vaccination of older adults and young children weakly dominating vaccination of older adults only and both strategies being highly cost-effective compared to use of unadjuvanted vaccine in the these groups (**Table 4**).

**Table 4:** Incremental cost-effectiveness of influenza vaccination strategies targeting children and older adults implemented in the Canadian population: no immunization of individuals aged 6–64.

Strategy	Vaccine efficacy	Cost ($ billion)^a^	QALY lost (million)^b^	Incremental cost per QALY gained ($)
Immunization of children and older adults with TIV	0.5 in children aged < 6; 0.2 in older adults	1.087	1.289	–
Immunization of children with TIV and older adults with ATIV	0.5 in children aged < 6; 0.4 in older adults	1.157	1.241	Weakly dominated^c^
Immunization of children and older adults with ATIV	0.875 in children; 0.4 in older adults	1.162	1.226	1190

Abbreviations: TIV, trivalent inactivated vaccine; ATIV, adjuvanted trivalent inactivated vaccine.

a 2009 Canadian dollars, discounted at 5% annually over a 10-year time horizon.

b Quality-adjusted life years lost, discounted at 5% annual over a 10-year time horizon.

c Immunization of older adults only with ATIV was economically attractive at $1424 per QALY, but the incremental cost-effectiveness ratio of immunizing *both* older adults and young children with ATIV was <$300 per QALY, indicating “extended dominance”.

### Sensitivity Analysis

**Figure 3:**
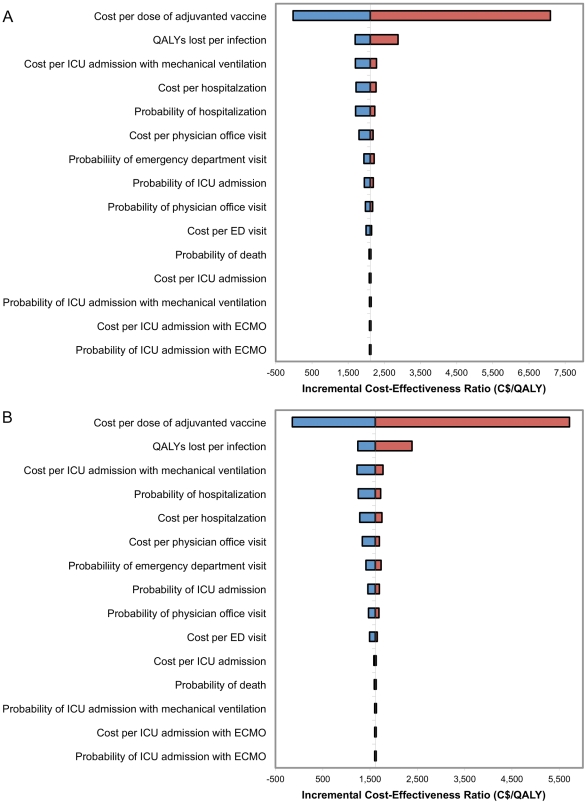
Tornado diagram comparing the relative importance of model parameters on estimated cost-effectiveness. Incremental cost-effectiveness ratios (ICER) are calculated relative to the use of unadjuvanted vaccine in the entire population when adjuvanted vaccine is used in (a) older adults and (b) older adults and young children. The vertical line corresponds to the base case value for each parameter, with the width of the bars indicating the uncertainty associated with each parameter. The blue segments of the bars correspond to parameter values that result in decreased estimates of cost effectiveness (with negative values corresponding to projected cost savings), while red segments indicate values that increase the base case cost effectiveness. The range of parameters considered in the analysis is described in **Table 2** and **File S1**.

The projected cost-effectiveness of introducing an adjuvanted vaccine in older adults (**Figure 3(a)**) or older adults and young children (**Figure 3(b)**) was most sensitive to estimates of the cost of adjuvanted vaccine and QALYs lost per infection, but still remained a highly cost-effective intervention in all scenarios. Using the best case set of parameter values, introduction of adjuvanted vaccine was projected to be cost-saving, saving $3350 and $3153 per QALY gained with use of ATIV older adults or older adults and young children, respectively, compared to use of TIV in the entire population. In the worst case scenario, use of ATIV was projected to cost $10,647 and $9472 per QALY gained with the older adults only and older adults and young children strategies, respectively, compared to the use of TIV only.

We estimated the vaccine efficacy in older adults at which use of adjuvanted vaccine was no longer a cost-effective strategy. When ATIV efficacy in older adults was 0.21 or greater (compared to the baseline estimate of 0.2 for TIV), use of ATIV was cost-effective, costing less than $50,000 per QALY gained. Similarly, when ATIV efficacy in children was greater than 0.51 (versus 0.5 for TIV), expanding the use of ATIV to include children was cost-effective (ICER $38,748.34) relative to the use of adjuvanted vaccine in older adults only. Similar results were observed when we excluded immunization of the population aged 6–64.

**Figure 4:**
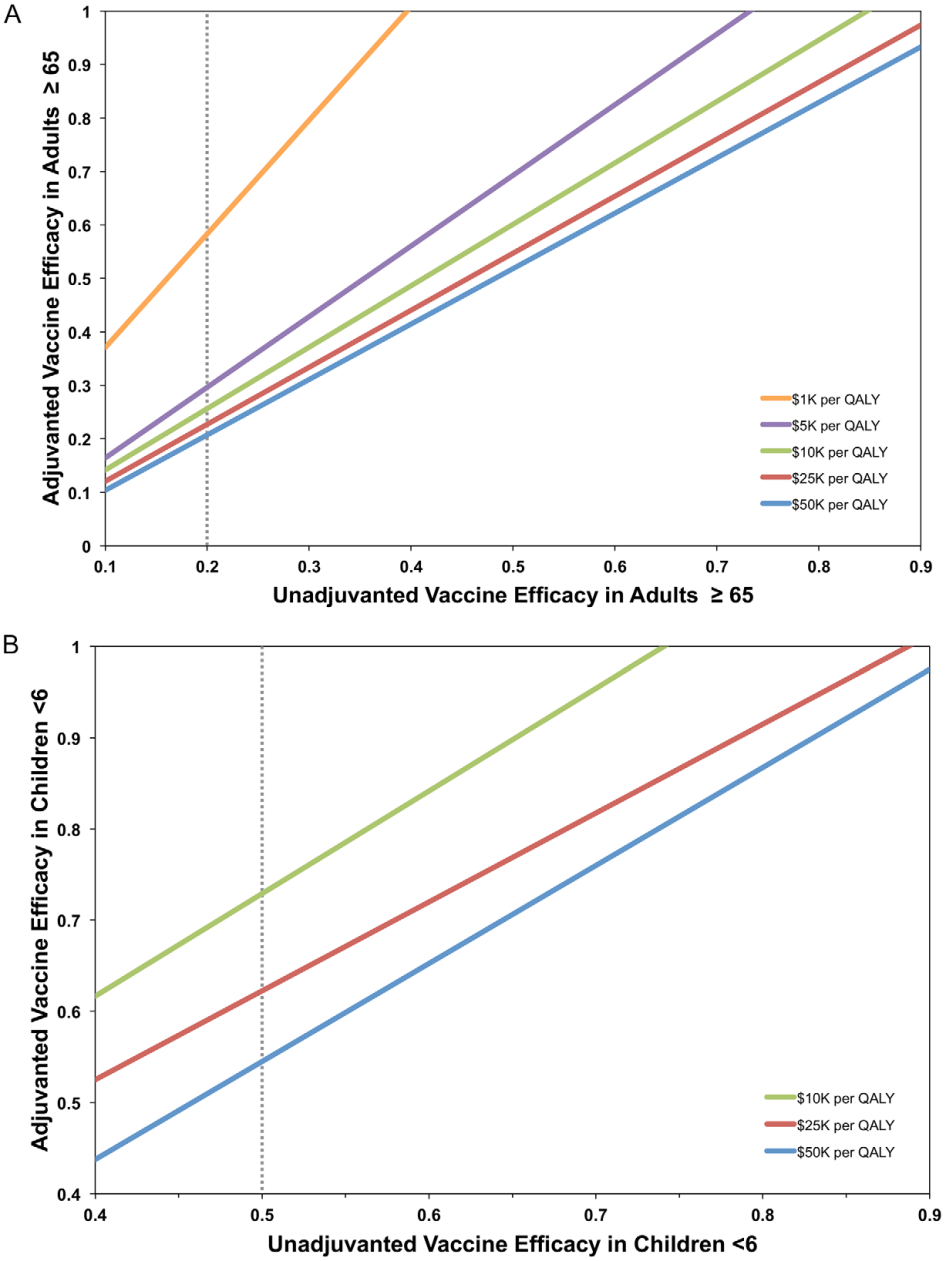
Vaccine efficacy values above which use of adjuvanted vaccine is no longer the preferred strategy. Thresholds were determined for different assumed unadjuvanted vaccine efficacies in (a) older adults and (b) young children, assuming different willingness-to-pay thresholds. Unadjuvanted vaccine efficacy used in base case scenarios is indicated by a dotted line.

Vaccine efficacy values at which use of ATIV was no longer the preferred strategy were evaluated for different willingness-to-pay thresholds. We calculated these thresholds for different assumed vaccine efficacies for TIV in older adults (**Figure 4(a)**) and young children (**Figure 4(b)**).

Assuming no difference in vaccine efficacy but enhanced duration of immunity following immunization with ATIV compared to TIV, use of ATIV was projected to be highly cost-effective when used in children and older adults. Specifically, when duration of vaccine-induced immunity with ATIV was 1.3 years (equivalent to that conferred by natural infection) compared to 1 year with TIV, the ICER was $6665 per QALY. When ATIV-induced immunity was modeled as more durable (1.3 years) and more effective than TIV in older adults and young children, the ICER was reduced to $882 per QALY.

## Discussion

Optimal control strategies for influenza continue to generate controversy among public health communicable disease control experts. To inform this debate, we developed a mathematical to project the impact and cost-effectiveness of a novel adjuvanted seasonal influenza vaccine in the Canadian population based on the best-available data. Use of ATIV in seniors and young children was projected to provide substantial health benefits, and to be cost effective, relative to currently used TIV. Although the impact of adjuvanted vaccine on absolute numbers of deaths was greatest in seniors at highest risk of fatal outcomes, we projected that it would also avert substantial numbers of hospitalizations in younger individuals. The incorporation of transmission into the model made it possible to project the gains in health and survival in age groups not receiving the adjuvanted vaccine; we projected that the use of adjuvanted vaccine in children, in particular, would markedly reduce hospitalizations in children and adults not targeted to receive adjuvanted vaccine. Such “herd effects” are consistent with effects demonstrated in recent randomized controlled trials, in which immunization of younger individuals protects the population as a whole [24], [25].

We incorporated costs and health utility weights, which have been used in prior published health economic analyses [18], [22], into our model to assess the economic attractiveness of replacing immunization of older Canadians and young children with adjuvanted vaccine. Proposed World Health Organization benchmarks suggest that programs be considered highly cost-effective if life years are purchased at a cost of less than per-capita gross domestic product [26], which in Canada is approximately $40,000. In our base case we projected that immunization of older adults with ATIV would be extremely cost-effective relative to the use of traditional TIV, even in the context of a universal influenza immunization program like that in effect in Ontario, which appears to have reduced mortality in the elderly indirectly, via prevention of transmission of influenza from younger to older individuals [17]. Cost-effectiveness was further enhanced when we eliminated the Ontario-style UIIP from the model, with the direct protection provided to older individuals by adjuvanted vaccine counterbalancing the loss of indirect protection accrued via immunization of younger adults. The relative novelty of adjuvanted influenza vaccines makes modeling challenging, given that the true values of vaccine efficacy parameters are not yet known with certainty; however, there is a growing body evidence supporting the contention that these vaccines are more effective in children and older adults than traditional unadjuvanted vaccines [9], [11], [27], [28]. Given the uncertainty in data inputs in the model, we subjected our projections to extremely wide-ranging sensitivity analyses and found them to be extremely robust; the use of adjuvanted vaccine was preferred in older individuals even when “best case” values (efficacy  =  0.5) were used for TIV and “worst case” (efficacy 0.51) values were used for ATIV. While this may appear surprising, the health and economic toll of influenza in older adults in typical influenza seasons is extremely high [29], [30], [31], [32]. Consequently, the direct protection provided by ATIV in this group translates into large health gains at low economic costs, even when the gap in effectiveness between vaccine types in older individuals is modeled as far smaller than would be expected based on the best available data [10]. Pediatric effectiveness data, being derived from a well-designed randomized controlled trial [9], [11], are subject to less uncertainty, but our projections of cost-effectiveness are nonetheless robust in the face of substantial variation in estimated efficacy in children.

While we assigned an efficacy of 20% to TIV in older adults in our base case, evidence for effectiveness of TIV in older adults is conflicting, with some studies reporting effectiveness as high as 50–60% [19], [33], while others fail to find any evidence of effectiveness when circulating strains do not match vaccine components, or when influenza epidemics are absent [20]. Furthermore, estimates of the impact of influenza vaccine on all-cause mortality in older individuals are implausibly large given levels of vaccine coverage seen in countries such as the United States, and the relatively limited proportion of deaths which are excess deaths during influenza season [34]. The apparent impact of influenza vaccination on mortality in non-influenza season has served to provide further evidence that effects attributed to influenza vaccination may in some cases represent a “healthy vaccinee effect”, with more robust elderly individuals being more likely to receive vaccination [35], [36]. Interestingly, the large observational study of ATIV that is the source of our base-case effectiveness estimates was subject to exactly the opposite limitation: in that study, older individuals with poor health status preferentially received ATIV (while their healthy counterparts received TIV), and the excess risk of hospitalization seen in these individuals was confined to the period *outside* influenza season [10], suggesting that the true relative efficacy of ATIV may be higher than we estimate in our base-case analysis.

Emerging data suggest that MF59-adjuvanted vaccines appear to confer cross-strain immune protection sufficiently robust to provide protection against drifted influenza strains, via generation of antibody and B-cell responses against a broader range of influenza antigens than is the case with unadjuvanted vaccine [12], [37], [38]. We project that enhanced durability of protection could make ATIV economically attractive even in the absence of increased effectiveness; further research is needed to evaluate the relative durability of effect of these vaccines.

Like any model-based evaluation of vaccine effectiveness and cost-effectiveness, our analysis has limitations. Our mathematical model includes simplifying assumptions and incorporates parameters values that are subject to uncertainty. Model calibration to existing data was used to reduce this uncertainty for some key parameters and wide-ranging sensitivity analyses were used to explore the impact of parameter uncertainty on our findings. We used a constant value for estimates of vaccine efficacy, although these values will vary from year-to-year, depending on match with circulating influenza strains. We excluded vaccine-related adverse events; although studies to date have not suggested elevated risks of serious adverse events associated with the MF59 adjuvant [39], immune adjuvants may result in unusual adverse event profiles [40], [41], [42], [43]. Ongoing surveillance and evaluation of vaccine-associated adverse event risks are warranted for this novel vaccine.

In summary, a mathematical model parameterized to represent the transmission of influenza in the Canadian population suggests that use of an adjuvanted trivalent influenza vaccine in seniors and young children is likely to be a highly cost-effective intervention, relative to the currently used unadjuvanted vaccine. These projections hold even under assumptions of very minor enhancements of vaccine efficacy associated with adjuvanted vaccines. Enhanced durability of vaccine-derived immunity may further enhance the economic attractiveness of this intervention.

## Supporting Information

File S1 **Supplementary appendix.**
(DOC)

Figure S1 **Model calibration to average excess influenza-attributable mortality.** Average influenza mortality was estimated using a smoothed time-series of average influenza-attributable mortality for the province of Ontario over seven influenza seasons, as described in the Methods section. Average reported age-specific vaccine uptake rates in Ontario for the time period under study (1997–2004) were used.
(TIFF)
