# Supplementary material for: Estimation of the Health Impact and Cost-Effectiveness of Influenza Vaccination with Enhanced Effectiveness in Canada
Source: PLoS One. 2011 Nov 14;6(11):e27420. doi: 10.1371/journal.pone.0027420 (PMC3215749; doi:10.1371/journal.pone.0027420)
Supplement: File S1 — Supplementary appendix. (DOC) [file pone.0027420.s001.doc]

**Supplementary Appendix 1**

*Mixing Patterns and Population Dynamics*

To explore how vaccination of different age groups would impact overall influenza morbidity and mortality and to enable the representation of more realistic contact patterns within and between age groups, we included age stratification with demographic information was obtained from 2006 Canadian census data . Mixing within and between age strata was based on a population-based prospective study of contact patterns in eight European countries . The birth rate was set equal to the death rate to maintain a constant population size and population distribution among age classes. Deaths occurred only in the oldest age group, with continuous aging through the age cohorts. Loss of immunity as a result of viral drift was incorporated using estimates contained in . Additional information on model parameter values, by age group, is presented in **Table 1**. Influenza seasonality was simulated by incorporating a cosine term into the model transmission parameter (*β*) , such that:

*β*(t) = *β*_1_*(1 + *β*_2_*cos(2*π*t))

The rate of infection at some time t was given as:


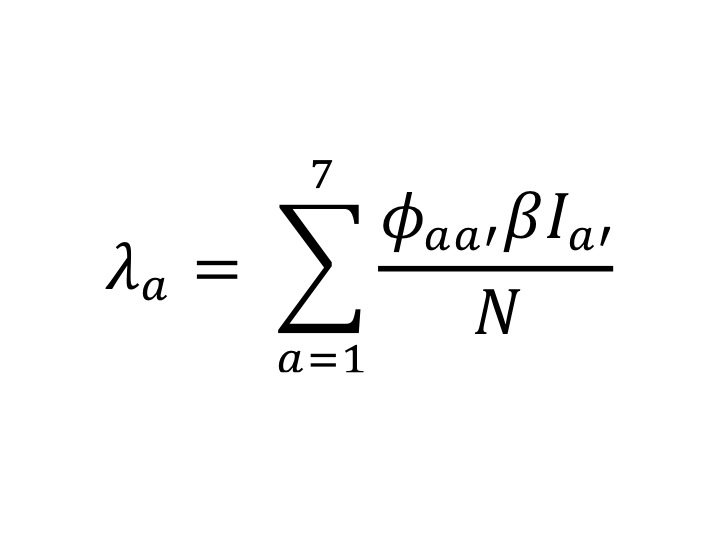


where *φ_aa’_* is the contact rate for infective individuals of age group *a’* (*I_a’_*) with susceptible individuals of age group *a*, *β* is the probability of transmission given contact (assumed to be independent of age), and *N* is total population size.

*Immunization*

Vaccination was modeled by removing a select number of individuals from the susceptible compartment immediately following administration of the vaccine. Vaccination occurred for a three-month period each year (beginning approximately 4 months prior to peak influenza activity, equivalent to starting a vaccination campaign in early October, with influenza activity peaking in late January), with the daily hazard of vaccination in each age group estimated as:

*H_V_* = *-ln*(1*-P_V_*)/t

where *H_V_* is the hazard of vaccination, *P_V_* is the cumulative probability of vaccination over time, and t is the duration of the vaccination “season”, assumed to be 3 months. For a given age group, *a*, with coverage P_v_, and vaccine efficacy VE, the proportion removed from the susceptible to the vaccinated compartment each vaccination season was S*_a_**P_v_*VE. We assumed that this group was fully protected against influenza infection, with the remaining fraction, S*P_v_*(1-VE) receiving no protection. Although this does not reflect the true situation, where most vaccinated individuals will receive some degree of protection, this approach has been used previously and has been demonstrated to provide a reasonable model of partial efficacy . This approach also allows for the modeling enhanced duration of immunity to future influenza vaccination among successfully vaccinated individuals.

*Calibration and Estimation of Baseline Burden of Disease*

A Serfling-type model that incorporated Fourier transform terms into Poisson regression models , was used to estimate “expected” influenza-attributable excess mortality. Calibration was performed using age-specific vaccination rates reported for Ontario . Calibration was used to derive estimates of the basic reproductive number, duration of immunity following naturally-acquired influenza infection, and vaccine efficacy in the population aged 6 to 64. Vaccine efficacy in adults aged ≥65 was set at 0.2 and in children under 6 vaccine efficacy was estimated to be 0.5. To estimate rates of illness and mortality that would be observed in the absence of vaccination, we removed influenza vaccination from the model.

*Effects of Enhanced Vaccine Efficacy in Children and Older Adults*

In base case analyses regarding infection risk in children aged <6 years, infection risk reduction with adjuvanted vaccine was conservatively estimated as 0.80 to 0.95 relative to non-vaccination (RR_AN_ = 0.05 to 0.20), while the risk reduction associated with adjuvanted vaccination was estimated as 0.70 to 0.80 (RR_AT_ 0.20 to 0.30) relative to trivalent vaccination in the same age group . The efficacy of trivalent influenza vaccination was defined as:

(RR_AN_/ RR_AT_) = (Risk_A_/Risk_N_)/(Risk_A_/Risk_T_)

= Risk_T_/Risk_N_ = RR_TN_

As such, we used estimates of efficacy of trivalent influenza vaccination in children ranging from 0 to 0.83, with a base case value of 0.5.

For base case analyses regarding infection risk in adults aged ≥65, the use of adjuvanted vaccine was estimated to reduce the risk of infection by 25% relative to trivalent vaccine ; assuming baseline efficacy of 0.2 for TIV (used in model calibration), this corresponded to a base case efficacy of 0.4 for adjuvanted for vaccine.

In the enhanced efficacy scenarios, we assumed the entire population was immunized at UIIP rates. Individuals aged 6-64 were immunized with trivalent influenza vaccine, with a vaccine efficacy of 0.9 in all scenarios. We evaluated the following strategies: (i) immunization of children and older adults with trivalent vaccine; (ii) immunization of children with trivalent vaccine and older adults with adjuvanted vaccine; and (iii) immunization of children and older adults with adjuvanted vaccine. We repeated the same scenarios in the absence of vaccination in the 6-64 age group.

*Estimation of Burden of Disease and Costs*

The age-specific impact of influenza on healthcare utilization, and cost, was estimated using the approach of Sander et al. and based on event probabilities as described by Kwong et al. . Briefly, costs were estimated as the product of probability of healthcare utilization events (i.e., outpatient clinic visits, emergency department visits, and hospitalizations) multiplied by the number of resource units associated with such events, and the unit-cost of such resources. These probability-weighted cost estimates represented the future stream of acute healthcare costs in individuals with incident influenza infection, and detailed estimates are presented FY 2009 dollars in the table below. Direct costs of vaccine administration and per-dose costs of maintaining a public vaccination program were based on estimates from Ontario’s UIIP. An adjuvanted influenza vaccine was used in Canada during the 2009 pandemic; the cost of this vaccine was approximately $8.00 per dose , or double the cost per dose of TIV used in the Ontario UIIP. We assigned an $8.00 cost per adjuvanted vaccine dose in the base case, with administration costs considered equivalent to those for TIV. A ten-year time horizon was used in the analysis, as this is the minimum amount of time that has been observed between influenza pandemics and also likely corresponds to a reasonable time-horizon for public health planners. We did not include pandemic years in the analysis. Future costs and health outcomes were discounted to present value at an annual rate of 5% .

Detailed breakdown of parameter values used in economic evaluation (from: Sander, B. et al., *Is a mass immunization program for pandemic (H1N1) 2009 good value for money? Evidence from the Canadian Experience.* Vaccine, 2010).

| **Item** | **Details** | | **Age group** | **Value** |
| --- | --- | --- | --- | --- |
| Probability of event |  |  |  |  |
|  | Physician services (conditional on symptomatic illness) |  |  |  |
|  |  | Office visits |  |  |
|  |  |  | All | 0.1 (0.05 to 0.2) |
|  |  | ED visits |  |  |
|  |  |  | All | 0.025 (0.01 to 0.05) |
|  |  | Hospitalization (conditional on symptomatic illness) |  |  |
|  |  |  | 0–4 | 0.00089 (0.00066 to 0.00166) |
|  |  |  | 5–19 | 0.00018 (0.00013 to 0.00033) |
|  |  |  | 20–64 | 0.00033 (0.00025 to 0.00062) |
|  |  |  | ≥65 | 0.00132 (0.00098 to 0.0024) |
|  |  | ICU (conditional on hospitalization) |  |  |
|  |  |  | 0–4 | 0.12 (0.06 to 0.24) |
|  |  |  | 5–19 | 0.11 (0.06 to 0.23) |
|  |  |  | 20–64 | 0.23 (0.11 to 0.46) |
|  |  |  | ≥65 | 0.16 (0.08 to 0.31) |
|  |  | ICU + mechanical ventilation (conditional on ICU) |  |  |
|  |  |  | All | 0.81 (0.65 to 0.9) |
|  |  | ICU + ECMO (conditional on ICU) |  |  |
|  |  |  | All | 0.04 (0.02 to 0.08) |
|  |  | Death (conditional on hospitalization) |  |  |
|  |  |  | 0–4 | 0.01 (0 to 0.01) |
|  |  |  | 5–19 | 0.02 (0.01 to 0.05) |
|  |  |  | 20–64 | 0.1 (0.05 to 0.21) |
|  |  |  | ≥65 | 0.19 (0.09 to 0.37) |
| Resource use |  |  |  |  |
|  | Physician services |  |  |  |
|  |  | Number of office visits |  |  |
|  |  |  | 0–4 | 1.17 (1 to 1.35) |
|  |  |  | 5–19 | 1.13 (1 to 1.26) |
|  |  |  | 20–64 | 1.16 (1 to 1.32) |
|  |  |  | ≥65 | 1.28 (1 to 1.56) |
|  |  | Number of ED visits |  |  |
|  |  |  | 0–4 | 1.23 (1 to 1.45) |
|  |  |  | 5–19 | 1.15 (1 to 1.29) |
|  |  |  | 20–64 | 1.26 (1 to 1.53) |
|  |  |  | ≥65 | 1.52 (1 to 2.03) |
|  | Hospitalizations – Resource intensity weight (RIW) |  |  |  |
|  |  | Ward |  |  |
|  |  |  | 0–4 | 0.79 (0.44 to 1.43) |
|  |  |  | 5–19 | 0.94 (0.44 to 2.27) |
|  |  |  | 20–64 | 1.48 (0.48 to 4.05) |
|  |  |  | ≥65 | 1.88 (0.6 to 5.02) |
|  |  | ICU, no ventilation |  |  |
|  |  |  | 0–4 | 5.21 (0.52 to 24.56) |
|  |  |  | 5–19 | 4.42 (0.44 to 22.32) |
|  |  |  | 20–64 | 2.82 (0.55 to 8.89) |
|  |  |  | ≥65 | 3.24 (0.72 to 9.84) |
|  |  | ICU and mechanical ventilation |  |  |
|  |  |  | 0–4 | 8.15 (0.6 to 30.85) |
|  |  |  | 5–19 | 8.17 (0.62 to 25.27) |
|  |  |  | 20–64 | 9.85 (0.8 to 30.07) |
|  |  |  | ≥65 | 9.22 (0.79 to 29.56) |
|  |  | ICU and ECMO |  |  |
|  |  |  | 0–4 | 25.5 (4.46 to 68.78) |
|  |  |  | 5–19 | 39.91 (3.26 to 123.07) |
|  |  |  | 20–64 | 15.83 (3.83 to 25.34) |
|  |  |  | ≥65 | 15.83 (3.83 to 25.34) |
|  | Hospitalizations – Physician services (claims/hospitalization) |  |  |  |
|  |  | Ward |  |  |
|  |  |  | 0–4 | 8.46 |
|  |  |  | 5–19 | 10.83 |
|  |  |  | 20–64 | 17.34 |
|  |  |  | ≥65 | 19.31 |
|  |  | ICU |  |  |
|  |  |  | 0–4 | 39.43 |
|  |  |  | 5–19 | 41.54 |
|  |  |  | 20–64 | 51.87 |
|  |  |  | ≥65 | 47 |
| Unit cost estimates ($) |  |  |  |  |
|  | Physician services |  |  |  |
|  |  | Office visit |  |  |
|  |  |  | 0–4 | 41.11 (28.55 to 64.05) |
|  |  |  | 5–19 | 36.3 (19.53 to 64.05) |
|  |  |  | 20–64 | 34.79 (19.53 to 61) |
|  |  |  | ≥65 | 40.21 (17.75 to 132.5) |
|  |  | ED visit (physician charge) |  |  |
|  |  |  | 0–4 | 51.98 (18.7 to 147.8) |
|  |  |  | 5–19 | 47.01 (21 to 73.9) |
|  |  |  | 20–64 | 47.15 (18.7 to 132.5) |
|  |  |  | ≥65 | 53.43 (17.1 to 132.5) |
|  |  | ED visit (non-physician cost per visit) |  |  |
|  |  |  | All | 181 |
|  | Hospitalization - Cost per RIW |  |  |  |
|  |  |  | All | 5339 |
|  | Hospitalization - Physician services (cost/claim) |  |  |  |
|  |  | Ward |  |  |
|  |  |  | 0–4 | 52.98 (11.05 to 147.8) |
|  |  |  | 5–19 | 49.47 (9.75 to 147.8) |
|  |  |  | 20–64 | 48.25 (8.65 to 132.5) |
|  |  |  | ≥65 | 43.4 (6.6 to 132.5) |
|  |  | ICU |  |  |
|  |  |  | 0–4 | 65.35 (6.6 to 192.45) |
|  |  |  | 5–19 | 62.58 (6.6 to 192.45) |
|  |  |  | 20–64 | 66.47 (6.6 to 192.45) |
|  |  |  | ≥65 | 63.1 (6.6 to 192.45) |
| Quality-adjusted life years |  |  |  |  |
|  | Influenza case |  |  |  |
|  |  |  | 0–4 | 0.98536 (0.9935 to 0.9779) |
|  |  |  | 5–19 | 0.98536 (0.9935 to 0.9779) |
|  |  |  | 20–64 | 0.98259 (0.99035 to 0.9755) |
|  |  |  | ≥65 | 0.97066 (0.97675 to 0.96514) |
|  | Death |  |  |  |
|  |  | Undiscounted |  |  |
|  |  |  | 0–4 | 71.54 |
|  |  |  | 5–19 | 62.2 |
|  |  |  | 20–64 | 34.38 |
|  |  |  | ≥65 | 2.85 |
|  |  | Discounted 5% |  |  |
|  |  |  | 0–4 | 18.53 |
|  |  |  | 5–19 | 18.15 |
|  |  |  | 20–64 | 15.14 |
|  |  |  | ≥65 | 2.41 |
