# Supplementary figures and images for: Estimation of the Health Impact and Cost-Effectiveness of Influenza Vaccination with Enhanced Effectiveness in Canada
Source: PLoS One. 2011 Nov 14;6(11):e27420. doi: 10.1371/journal.pone.0027420 (PMC3215749; doi:10.1371/journal.pone.0027420)

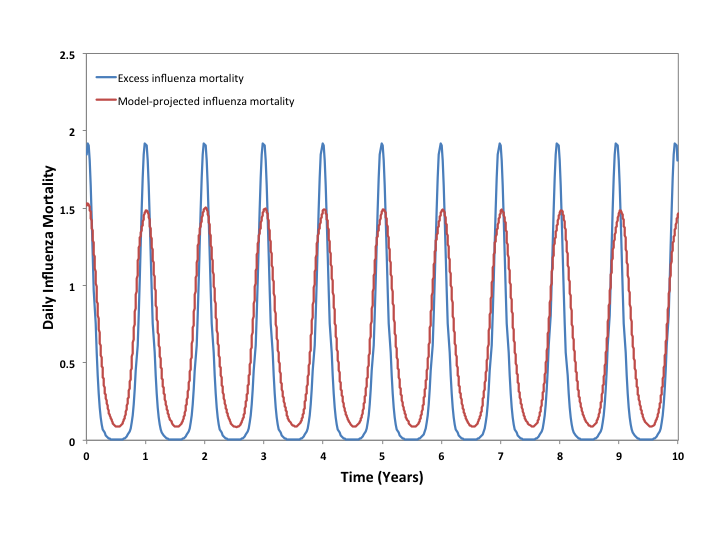

Supplement: Figure S1 — Model calibration to average excess influenza-attributable mortality. Average influenza mortality was estimated using a smoothed time-series of average influenza-attributable mortality for the province of Ontario over seven influenza seasons, as described in the Methods section. Average reported age-specific vaccine uptake rates in Ontario for the time period under study (1997–2004) were used. (TIFF) [file pone.0027420.s002.tiff]
